# Dynamic Data Streams for Time-Critical IoT Systems in Energy-Aware IoT Devices Using Reinforcement Learning

**DOI:** 10.3390/s22062375

**Published:** 2022-03-19

**Authors:** Fawzy Habeeb, Tomasz Szydlo, Lukasz Kowalski, Ayman Noor, Dhaval Thakker, Graham Morgan, Rajiv Ranjan

**Affiliations:** 1 School of Computing, Newcastle University, Newcastle upon Tyne NE1 7RU, UK; graham.morgan@ncl.ac.uk (G.M.); raj.ranjan@ncl.ac.uk (R.R.); 2 Alkamil College of Computer Science, University of Jeddah, Jeddah 21959, Saudi Arabia; 3 Institute of Computer Science, AGH University of Science and Technology, 30-059 Krakow, Poland; tomasz.szydlo@agh.edu.pl (T.S.); lukowalski97@gmail.com (L.K.); 4 College of Computer Science and Engineering, Taibah University, Madinah 42353, Saudi Arabia; anoor@taibahu.edu.sa; 5 Department of Computer Science, University of Bradford, Bradford BD7 1DP, UK; dhavalkumar.thakker@gmail.com

**Keywords:** osmotic computing, Internet of Things, reinforcement learning

## Abstract

Thousands of energy-aware sensors have been placed for monitoring in a variety of scenarios, such as manufacturing, control systems, disaster management, flood control and so on, requiring time-critical energy-efficient solutions to extend their lifetime. This paper proposes reinforcement learning (RL) based dynamic data streams for time-critical IoT systems in energy-aware IoT devices. The designed solution employs the Q-Learning algorithm. The proposed mechanism has the potential to adjust the data transport rate based on the amount of renewable energy resources that are available, to ensure collecting reliable data while also taking into account the sensor battery lifetime. The solution was evaluated using historical data for solar radiation levels, which shows that the proposed solution can increase the amount of transmitted data up to 23%, ensuring the continuous operation of the device.

## 1. Introduction

The Internet of Things is a concept beginning to be a natural element of human development and technological progress. IoT devices are used in many areas of everyday life, including smart homes, factories and cities [1]. IoT devices are also used in time-critical systems, i.e., where it is essential to obtain data processing results in the shortest possible time [2]. Examples of such systems are various solutions used during natural disasters, such as fires or floods. The key factor of such systems is the processing of up-to-date, non-delayed data from sensors installed in IoT devices. To achieve this, devices should be ready to transmit a data stream with necessary requirements.

Unfortunately, transferring a significant amount of data from sensors is associated with high demand for energy to make the measurements and then send the data to the edge and computing clouds. However, this can be difficult to achieve for IoT devices with limited computing and power resources. Especially when they are powered by renewable energy sources such as solar energy. However, the device can respond to changes in the availability of renewable energy by changing the frequency of collecting and transmitting measurement data. The paper proposes dynamic data streams, which can be changed to consume the device’s available resources accordingly.

Nevertheless, dynamic data streams in time-critical systems are related to the intelligent management of their parameters. Therefore, we propose to use the concept based on autonomic computing [3] to manage their parameters through dedicated management agents that can monitor and plan adaptation actions. It is also important that the purpose of device adaptation may depend on the system’s operating goal. For example, in a flood risk situation, the system should work with the most up-to-date data possible, paying less attention to maintaining the system’s lifetime. On the other hand, during normal operation, the system should strive to maintain as long a lifetime as possible to prepare for emergency situations. The implementation of the autonomic computing concept in IoT devices is complex since they have very limited resources. Therefore, we propose the usage of cooperating osmotic agents associated with devices and the edge datacenters [4]. The agents operating on the devices send data regarding the device operation, e.g., battery level, current configuration, while the edge agent plans device reconfiguration actions, which are then sent in response to be executed on the devices.

The agent’s logic could be implemented in the form of decision rules specifying actions that will be performed in specific situations. However, it would require having a particular model of the device and the environment in which it works. Therefore, in the paper we propose to implement the agent’s logic based on reinforcement learning. It is used in systems where, based on the observation of the system operation and the actions taken, their effectiveness in the form of a reward can be assessed. In summary, the following are the paper’s primary contributions:

- we formulated the limitation of the power resources problem in the IoT device,
- we proposed reinforcement learning-based dynamic data streams for time-critical IoT systems in energy-aware IoT devices,
- we evaluated our proposed approach performance using a levee monitoring system in river flood scenario.

The paper is organized as follows. The second section deals with an overview of state of the art. The third section discusses the motivation, while fourth describes the formal model and problem definition. Section five presents the proposed concept based on reinforcement learning, which is then evaluated in Section six. Finally, the paper is summarized and future work is discussed.

## 2. Related Work

Adaptation of IoT devices with the help of reinforcement learning (RL) is widely described in the literature. In [5] different cases of RL applied to IoT ecosystems are presented. IoT devices can use adaptation mechanisms in different layers of the systems, e.g., a smart vehicle, in the perception layer, can decide on velocity, driving direction, or avoiding obstacles. In the application layer, on edge/cloud servers, decisions about scheduling tasks, caching data or resources of virtual machines allocation can be made. Finally, RL algorithms can control the bandwidth or rate of data sent in the network layer.

RL algorithms can be applied to improve outcomes of sensing coverage tasks [6]. For example, many sensors may cover the most extensive possible area maintaining low battery consumption. Basically, the large area coverage by the single sensor, results in substantial battery consumption. In multi-agent systems, where each device communicates with another, RL can be used to optimise device operations for sensing applications and decrease the overall battery consumption. For that purpose, Q-Learning algorithm can be modified by e.g., distributed value function [7] improving up the learning process in a distributed environment.

Authors in [8] compare three different approaches to maintaining the highest possible amount of data transferred from an IoT device with limited battery capacity. Two of them are online/offline optimisation which assumes that the upcoming energy and state of the environment is known. In contrast, the RL based approach, which is more likely to be held in real life scenarios, knows about energy and system state only casually. Results show that with time, the learning-based approach yields results compared to those using optimisation-based approaches. In [9], RL is used in the case of many battery powered user equipment communicating via limited access channels to the single base station. The goal was to maintain the highest possible bandwidth of data sent from equipment to the base station while keeping the device battery alive. In each time slot, the station receives state information from selected equipment. Then such a state is used to choose an action based on learnt policy, and then such action is broadcasted to the user equipment. As the action and state spaces were large, the Deep RL algorithms based on Long Short-Term Memory (LSTM) was used to estimate Q-values.

In mobile edge computing (MEC) RL can be used for adapting processor frequency [10]. In such a scenario, an RL agent is implemented on an edge server in order to reduce processing time for incoming requests. When a request arrives, the edge server checks the current state of CPU loads and battery and decides whether such a request should be processed at all. If accepted, the CPU frequency should be set to a higher operational frequency, requiring more energy resources. Experiments show that the server learns how to handle requests of different sizes in different states (e.g., battery state) by using RL. This yields better results than rule based methods, e.g., best/worst fit approaches or other learning solutions such as sliding windows.

A combination of RL with LSTM neural networks can be utilised for scenarios when an RL agent must decide whether sensors have to be turned on to sense actual data or the value can be predicted based on the historical measurements [11]. It is used to preserve balance between energy consumption and the accuracy of the measurements. Both the RL agent and LSTM network are pretrained and two approaches are compared-model-free and model-based RL algorithms. Experiments show that such algorithms allow turning off sensors when predictions are accurate enough to preserve battery lifetime.

## 3. Motivation

**Figure 1:**
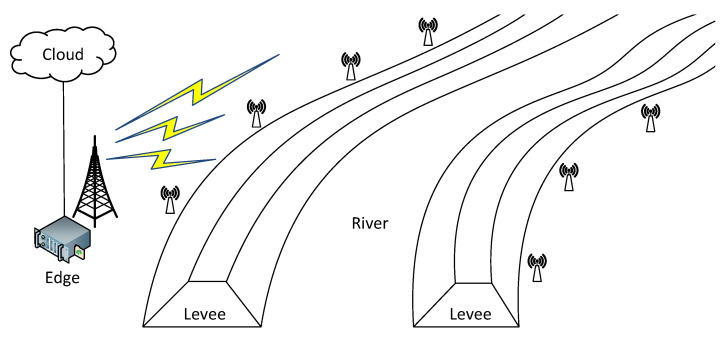
Levee monitoring system.

Consider the system for monitoring the condition of the levees built along the river, as shown in Figure 1. Its purpose is to detect situations that may result in the breakage of the levee during a flood and cause significant material and human losses.

IoT devices are located along the river and measure the physical parameters of flood barriers, including temperature, humidity and its displacement. The sensor data streams are then preprocessed in the edge station located in close proximity. The aggregated results are then sent to the cloud datacenter for further analysis. Communication between devices and the edge server is carried out using the wireless network.

The size of the data stream from sensors depends on the frequency with which the devices take measurements. However, increasing the amount of data transferred results in an increased demand for energy [12]. The devices are self-powered, thanks to the fact that they are equipped with photovoltaic panels and rechargeable batteries.

In the case of time-critical systems, it is necessary to send data as frequently as possible because, in the event of a flood hazard, actual sensor data are required [13]. Unfortunately, this can lead to a complete battery discharge, causing the device to be inactive until the next day and the next recharging cycle from soler panels. In a situation where there is no risk of damage to the embarkment, too frequent data transmission from sensors results in deep discharging cycles of the batteries, which contributes to the rapid degradation of their capacity and is undesirable.

The research problem concerns the adaptive management methods of the data stream from sensors in the presented class of systems. The research considers two scenarios. The first involves the operation of the IoT system under normal operating conditions. In this case, the purpose of adaptation is to ensure the longest possible operation of the system on the batteries and to reduce the complete discharge cycles. The second assumes the operation of the system during exceptional situations, requiring constant and frequent monitoring of the environment and sending data from sensors.

## 4. Formal Model

We begin by presenting the required definition and system description to represent our research problem in Section 4.1. We formulate our problem using these definitions (Section 4.2). The Abbreviations part lists all of the notations that were used in the paper.

### 4.1. System Description and Definition

The infrastructure system *X*, which is represented as a quintuple 〈O,PV,D,E,C〉. *O* is a set of Osmotic Agents that respond to communication between the devices and is denoted by $O_{o}$ = $\left\{id_{o}\right\}$, where $id_{o}$ represent the identifier of the Osmotic Agents $O_{o}$. PV is a set of Photovoltaic panels located in each IoT device $D_{i}$ and is denoted by $PV_{p}$ = $\left\{id_{p}\right\}$, where $id_{p}$ represent the identifier of the Photovoltaic panels $PV_{p}$. *D* is a set of IoT devices $D_{i}$ and is denoted by $D_{i}$ = $\left\{id_{i},\delta_{i},b_{i},r_{i},o_{i}\right\}$, $id_{i}$ represents the identifier of the IoT device $D_{i}$, $\delta_{i}$ represents the sensing rate of IoT device $D_{i}$, so, each IoT device observes its surroundings continuously over a given time interval, $b_{i}$ represents the battery of IoT device $D_{i}$, $r_{i}$ represents the renewable energy from the Photovoltaic PV panels, $o_{i}$ represents the osmotic agent of the IoT device $D_{i}$. *E* is a set of edge devices $E_{e}$, each $E_{e}$ is represented as $E_{e}$ = $\left\{id_{e},h_{e}\right\}$. Where $id_{e}$ and $h_{e}$ represent the identifier and the set of host machines $h_{e}^{1}$, $h_{e}^{2}$, … for the edge device $E_{e}$, respectively. *C* is a set of cloud data centres $C_{c}$, and is denoted by $C_{c}$ = $\left\{id_{c},h_{c}\right\}$ where $id_{c}$ is the identifier of the datacentre and $h_{c}$ is the set of host machines $h_{c}^{1}$, $h_{c}^{2}$, … for the cloud data center $C_{c}$, respectively.

An IoT application $A_{i}$ is defined as a directed acyclic graph (DAG) of microservice $A_{i}=\left\{A_{i}^{\mu_{1}},A_{i}^{\mu_{2}},...\right\}$ in which each $A_{i}^{\mu_{j}}$ represents a microservice to be execute. Each $A_{i}^{\mu_{j}}$ has its own set of software (SW), hardware (HW), and quality of service (Q) requirements. The combined requirements $R(A_{i}^{\mu_{j}})$ for a microservices are shown in Equation (1).

(1) $$R\left(A_{i}^{\mu_{j}}\right)={SW}^{\mu_{j}}+{HW}^{\mu_{j}}+Q^{\mu_{j}}$$

In Equation (2), the total requirements of any application $A_{i}$ is given by the sum up the requirements of all the microservices.

(2) $$R\left(A_{i}\right)=\sum_{\forall j}R\left(A_{i}^{\mu_{j}}\right)$$

Data are generated by IoT devices $D_{i}$ on a regular basis. The IoT device is treated as a passive entity, which means it does not handle data and instead sends it to the edge device. Each IoT device $D_{i}$ have a battery $b_{i}$ and a Photovoltaic panel $PV_{i}$ that will recharge the IoT device $D_{i}$ battery $b_{i}$ continuously. The total battery capacity $B_{total}$ is computed as given in Equation (3).

(3) $$B_{total}=b_{avl}+PV_{avl}$$

where $b_{avl}$ is the IoT device $D_{i}$ available battery capacity, and $PV_{avl}$ is the IoT device $D_{i}$ available Photovoltaic panel charging capacity. When the IoT device generates the data from the surrounding environment and sends it to the edge datacenter $E_{e}$, that process will consume the battery. So, to calculate the overall battery consumption BC for each IoT device using Equation (4).

(4) $$\mathrm{BC}=\frac{1}{s_{r}}\cdot t_{r}$$

where the $s_{r}$ is sensing rate of the environment and $t_{r}$ is draining rate of sending the data to the edge datacenter $E_{e}$.

### 4.2. Problem Definition

**Definition** **1.** *Given an infrastructure X={O,PV,D,E,C} and a set of IoT applications $A=\{A_{1},A_{2},...\}$, a suitable deployment solution $\Delta_{m}$ is defined as a mapping for $A_{i}\in A$ to X ($\Delta_{m}:A_{i}\to X\forall A_{i}$) if and only if:*

*1*. *$\forall A_{i}^{\mu_{j}}\in A_{i}$, $\exists (A_{i}^{\mu_{j}}\to v_{h})$ where, $h\in \{h_{e}\cup h_{c}\}$*
*2*. *$\forall A_{i}^{\mu_{j}}\in A_{i}$, if $A_{i}^{\mu_{j}}\to v_{h}$, then ${HW}^{\mu_{j}}⪯v_{h}^{HW}$ and ${SW}^{\mu_{j}}⪯v_{h}^{SW}$*
*3*. *$\sum_{\mu_{j}}{HW}^{\mu_{j}}\leq v_{h}^{HW}$ and $\sum_{\mu_{j}}{SW}^{\mu_{j}}\leq v_{h}^{SW}$*

All the requirements to find a suitable deployment solution are considered in the definition given above. Requirement 1 indicates that a mapping between $A_{i}^{\mu_{j}}$ and a virtual environment $v_{h}|h\in \left\{h_{e}\cup h_{c}\right\}$ must exist for every microservice belonging to the IoT application $A_{i}$. Requirement 2 confirms that the hardware and software requirements of the microservice must be satisfied by $v_{h}$ if a microservice $A_{i}^{\mu_{j}}$ is deployed to a virtual environment $v_{h}$. Finally, requirement 3 limits the number of microservices a virtual environment can execute at any time *t*.

The primary goal of this study is to find the best solution for all applications $A_{i}$ such that the overall battery consumption ${\mathrm{BC}}_{A_{i}}$ is minimum. As given these requirements, we can represent the problem as shown below.

(5) $$\begin{array}{c} \mathrm{minimize}\;{\mathrm{BC}}_{A_{i}}+\mathrm{minimize}\;s_{r}^{i}\\ \;\;\;\;\;\mathrm{subject}\mathrm{to}:\\ \forall i\in A_{i},\forall j\in \mu_{j}\;\exists \left(A_{i}^{\mu_{j}}\to v_{h}\right) \end{array}$$

The constraint states that all of the application’s microservices $A_{i}^{\mu_{j}}$ must be executed in a virtual environment (Equation (5)).

## 5. Osmotic Agents with RL

In the proposed solution, we leverage the osmotic agents [4] concept. Each device has an agent associated with it that manages the device’s resources. In a classic approach to RL, it is assumed that there is an agent-environment interaction in which there is a critic who can evaluate the actions taken. However, the proposed solution assumes two environments—one internal that is a shadow representation of the device and the other external that is a real device. First, the state of the internal environment is updated based on observation of the external device. Then, the assessment of the actions taken on external device is carried out based on the state of the internal environment.

We also assume that in the case of a network of IoT devices forming a sensor network, they are functionally similar and operate analogously. In other words, they are independent, but with a similar state distributions. This means that the internal environment represents a generic IoT device that is part of the system, and the knowledge update process may include observations from a set of devices.

**Figure 2:**
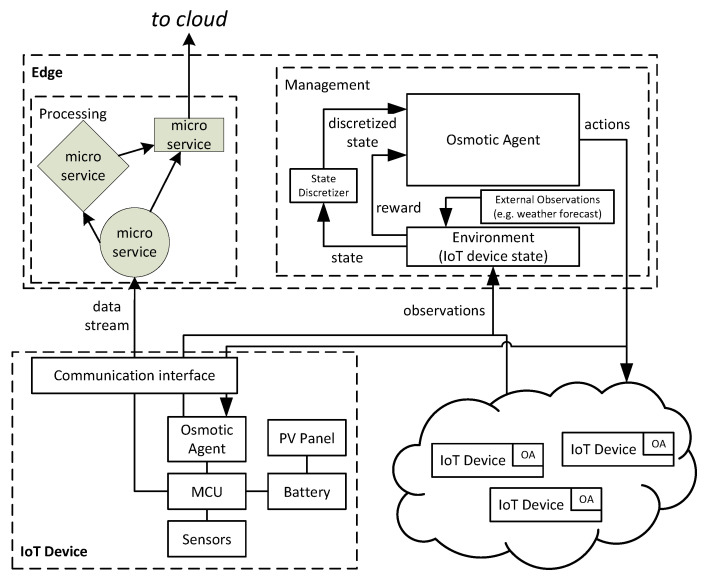
System architecture.

In the solution as presented in Figure 2, agents of different devices can communicate and create a multi-agent system. Due to the fact that the IoT device has limited computing and memory resources, the logic of the device adaptation is controlled by an agent running in the edge datacenter.

The internal environment used by the edge agent can be enriched with additional information from external sources. In our case, it is information about the weather forecast and forecast cloud cover for the current and next day.

### 5.1. QLearning Algorithm

We decided to use the classic Q-Learning algorithm to implement the RL logic of the agents managing the sensing rate. It is a model-free algorithm that learns the value of an action performed in a particular state. In our solution, the actions that can be performed on the device are the same as choosing the sensing rate, i.e., *A* = $\left\{s_{r}\right\}$. Hence, the function Q is defined as:

(6) Q:S×A→R

Updating the value of the Q function is done using the Bellman function as an iterative update using the weighted average of the old and new values:

(7) $$Q^{new}\left(s_{t},a_{t}\right)=Q\left(s_{t},a_{t}\right)+\alpha \cdot \left[r_{t}+\gamma \cdot \underset{a}{argmax}Q\left(s_{t+1},a\right)-Q\left(s_{t},a_{t}\right)\right]$$

The α parameter is responsible for the learning rate, i.e., how much new values during learning affect updating the current values. The γ parameter is responsible for the discount factor, i.e., how important long-term rewards are compared to short-term ones. Parameter values influence the learning process and are application dependent. Typically α=0.1 and γ=0.8 are assumed.

### 5.2. State Discretization

The number of possible states representing the environment requires a discussion. Initial analysis indicates that an RL problem with ample state space can be solved using DeepRL [9] methods. However, in the solution, we decided to limit the space states through their discretization, which is justified in reducing the resources needed for training the algorithm and implementation in real IoT devices. The possible states values for the presented problem are presented in Table 1.

### 5.3. Reward Function

The RL algorithm is based on the value of the reward obtained in response to the chosen actions. In our system, the reward function is a weighted average of two factors, where the β parameter determines the weight:

(8) $$r_{i}=\left\{\begin{array}{cc} \beta \cdot b_{i}+\left(1-\beta \right)\cdot \frac{min(s_{r}^{i})}{s_{r}^{i}} & if\;b_{i}⩾0.05\\ 0 & if\;b_{i}<0.05 \end{array}\right.$$

The first component concerns the device’s battery level and assumes values in the range [0;1]. The second component involves the sensing rate of the device. The more often the device collects data, the higher the value is. The second component also takes values in the range [0;1].

In the case of the discussed systems, the amount of data transferred from the device is important, but the more important issue is to prevent the situation of a complete discharge of the batteries. Therefore, the reward function is 0 if the device has a critical battery level of less than 5%, and the β parameter was set to 0.2 to include battery level in the reward function.

We have set the critical battery level as 5% due to the possible inaccuracy of the battery capacity measurement and the potential need for a safe device’s system shutdown. Therefore, we assumed that the RL agent receives a penalty if the battery level reaches the indicated value.

## 6. Evaluation

The solution was assessed using the *IoT-SimOsmosis* [14] simulator extended with a module enabling renewable energy analysis from photovoltaic panels. We assumed that the IoT devices monitor the dyke temperature, and its specification is presented in the Table 2. The simulation was carried out for historical data of solar radiation levels in 2016 obtained from the PVGIS database.

We have conducted experiments with various device management profiles regarding sensor data streams, including both constant and adaptive ones based on RL. The results achieved will be discussed in the following subsections.

### 6.1. Constant Data Streams

**Figure 3:**
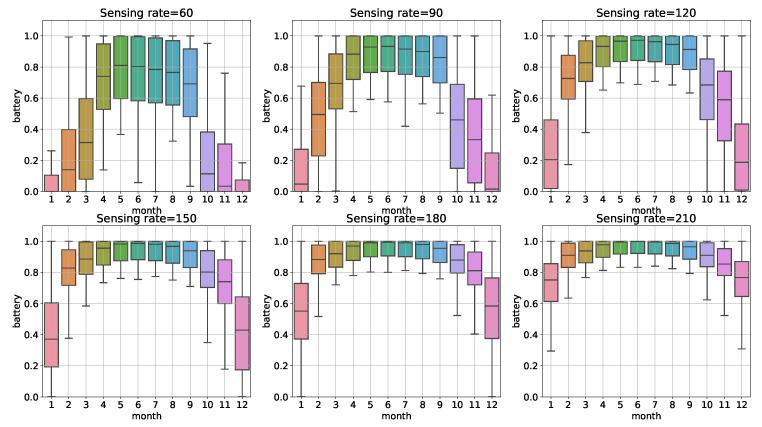
Battery levels of the device for various constant sensing rates. Colors of the boxes are related to the mean value of battery level.

In the case of constant management profiles, it was assumed that the device had a constant sensing rate value of 60 s, 90 s, 120 s, 150 s, 180 s and 210 s, respectively. We observed changes in the device’s battery levels during the experiment. The results grouped by months are presented in the Figure 3. We also counted during how many days in a year the device completely discharged the batteries. The results are presented in the Table 3. For measurements performed every 60 s, there were 166 days a year that the sensor stopped working due to a lack of energy, while the mean battery level was 47%. Most often, such situations occur during winter and spring seasons where solar radiation is lower than during summer periods. On the other hand, with measurements taken every 210 s, the device ran all year round without interruption having 89% of battery on average.

One of the solutions to assure frequent sensing is to oversize the size of the PV panels and the battery capacity, but this raises costs and is not very economical. However, we propose dynamic data stream management for the IoT device to solve this problem. The goal is to manage IoT devices to avoid a situation where the devices stop working due to a lack of energy and provide relevant sensor data required by the flood predictions.

### 6.2. Dynamic Data Streams

**Figure 4:**
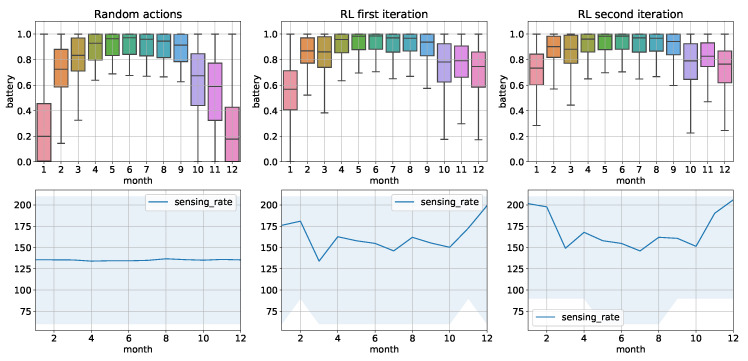
Battery levels and the selected sensing rates for the devices in RL based data stream management. Blue area represents *min* and *max* values of sensing rate, while the chart represent *mean* sensing rate value for the particular month.

In the case of dynamic data streams, we assumed that the system should learn online which of the actions taken receive the greatest reward during its operation. However, too much exploration may result in unexpected operation of the device. It can be observed in the example where random management actions, i.e., change in sensing rate, affect the operation of the device, as shown in Figure 4. As a result, there were 57 days during which the device stopped working due to battery discharge.

Therefore, the exploration process was limited to a random generation of the Q table during system initialization. As a result, the initial adaptation actions taken by the device were random, which allowed for state exploration. It is especially visible in the Figure 4 for the beginning months of the year (exploration resulting from a random Q table) where battery was discharged. During the ending months of the year, the system has already developed an adaptation policy and thus preventing battery discharge.

The evaluation was continued for the same historical data and the system was still carrying out the learning process. Still, the actions taken only improved the previously used policy in this case. As a result, continuous operation of the device was achieved throughout the year, with an average sensing rate of 170 s. During this time, the batteries in the device were not discharged and there was a 23% increase in the amount of data sent from the device compared to the constant 210 s profile as presented in Table 3.

## 7. Summary and Future Work

In the paper, we introduced the concept of dynamic data streams that change the data transfer rate according to the available energy resources. Thanks to the use of reinforcement learning, it is possible to adapt to the expected amount of energy obtained from renewable energy sources—from photovoltaic panels.

In the evaluation, we compare two different methods of managing data streams from IoT devices. In the simplest case for constant data streams, the user selects their parameters. If it is important to optimize battery consumption, the user can choose the highest possible sensing rate, i.e., sending data as rarely as possible. On the contrary, if it is essential to obtain detailed data about the monitored environment, the user can choose to transmit the data as often as possible. In this case, however, it may result in a complete discharge of the device.

In the second case, the proposed dynamic data streams represent the tradeoff between the described operational modes. It employs the Q-Learning algorithm to adjust the data transport rate based on the amount of renewable energy resources available, to ensure reliable collecting data while also taking into account the sensor battery lifetime. The solution was evaluated using historical data for solar radiation levels, which shows that the proposed solution can increase the amount of transmitted data up to 23% ensuring the continuous operation of the device.

Discussed data stream operation modes, i.e., highest sensing rate, minimal battery consumption and RL based dynamic one, can be activated depending on the user’s requirements and the intended purpose of the IoT system.

As future work, the development of the presented works can be twofold. The first aspect concerns the possibility of cooperation of the devices themselves while gaining experience and knowledge in device management. Then, RL agents can operate independently on each of the devices and exchange messages containing the type of action taken in a specific system state and possibly the achieved reward. The second aspect includes domain analysis of the monitored environment in which the devices forming the sensor network are placed. It is then possible to selectively monitor the environment so that devices take measurements alternately instead of simultaneously.

## Figures and Tables

**Table 1:** Discretized states used in the RL algorithm.

Observation	Number of States	State Discretization
Today Forecast	3	*cloudy; partly cloudy; sunny*
Next Day Forecast	3	*cloudy; partly cloudy; sunny*
Month	3	{1,2,11,12}{3,4,9,10}{5,6,7,8}

**Table 2:** IoT device specification used in the evaluation.

Device Type	Battery Capacity	Initial Energy	Battery Voltage	Solar Panel	Charging Current
Temperature Sensor	3000 mAh	2000 mAh	3.7 V	10 W	500 mA

**Table 3:** Data stream management profiles used in the evaluation.

Method	Mean Sensing Rate	Low Batt Days	Mean Batt Level
Constant 60 s	60 s	166	47%
Constant 90 s	90 s	105	62%
Constant 120 s	120 s	57	72%
Constant 150 s	150 s	26	79%
Constant 180 s	180 s	5	85%
Constant 210 s	210 s	0	89%
Random actions	135 s	57	72%
RL first iteration	162 s	8	83%
RL second iteration	170 s	0	85%

## Data Availability

Not applicable.

## Abbreviations

The following abbreviations are used in this manuscript:

**Symbol**	**Description**
*X*	The system infrastructure
*O*	A set of Osmotic Agents
PV	A set of Photovoltaic panels
*D*	set of IoT devices
*E*	A set of Edge devices
*C*	A set of Cloud data centers
*h*	A set of host machines
*v*	Virtual environment
δ	The data rate of IoT device
*b*	IoT device battery
*r*	The renewable energy from the Photovoltaic panels *P*
A	An IoT application
SW	A software
HW	A hardware
Q	Quality of Service
R	Requirements
$B_{total}$	The total battery capacity
$b_{avl}$	the IoT device $D_{i}$ available battery capacity
$P_{avl}$	the IoT device $D_{i}$ available Photovoltaic panel *P* charging capacity.
BC	The overall battery consumption
$s_{r}$	Sensing rate of the environment
$t_{r}$	A draining rate of sending the data to the edge datacenter *E*
*Q*	Q function
*A*	An actions
*S*	A states
R	A reward
α	Learning rate
γ	Discount factor
β	The weight
